# Associations of Mediterranean, DASH, and MIND diet adherence with metabolic parameters in individuals with type 2 diabetes mellitus: a cross-sectional study

**DOI:** 10.1007/s11845-026-04415-x

**Published:** 2026-04-29

**Authors:** Nurefşan Konyalıgil Öztürk, Tuba Taslamacıoğlu Duman

**Affiliations:** 1 Faculty of Health Sciences, Department of Nutrition and Dietetics, Bolu Abant Izzet Baysal University, Bolu, Turkey; 2 Faculty of Medicine, Department of Internal Medicine, Bolu Abant Izzet Baysal University, Bolu, Turkey

**Keywords:** Cardiometabolic risk factors, DASH diet, Glycemic control, Mediterranean diet, Type 2 diabetes mellitus

## Abstract

**Background:**

The aim of this study was to examine the associations between adherence to theDietary Approaches to Stop Hypertension (DASH) diet, the Mediterranean diet, and the Mediterranean-DASH Intervention for Neurodegenerative Delay (MIND) diet and metabolic parameters in individualswith Type 2 diabetes mellitus (T2DM).

**Methods:**

This cross-sectional study was conducted with 90individuals with T2DM. Participants completed a questionnaire form containing sociodemographiccharacteristics, and their anthropometric measurements, biochemical parameters, body compositionanalyses, Visceral Adiposity Index, and nutritional status were evaluated.

**Results:**

According tomultivariate regression analyses, each 1-point increase in the MIND diet score was found to be inversely +associated with LDL-cholesterol (β = − 0.011), triglycerides (β = − 0.010), and Visceral Adiposity Index (β = − 0.387). Each 1-point increase in the Mediterranean diet score was inversely associated with Body Weight (β = − 0.028), Waist Circumference (β = − 0.058), Visceral Fat Percentage (β = − 0.139), Fasting Blood Glucose (β = − 0.016), and Triglycerides (β = − 0.014). Each 1-point increase in the DASH dietscore was associated with a decrease in fasting blood glucose (β = − 0.007) and triglyceride levels (β =− 0.005; *p*< 0.01).

**Conclusion:**

It has been observed that adherence to dietary patterns is associatedwith metabolic parameters in individuals with type 2 diabetes, and adherence to the Mediterranean diethas been found to be associated with a greater number of metabolic parameters compared to otherdietary patterns.

**Graphical abstract:**

**Figure:**
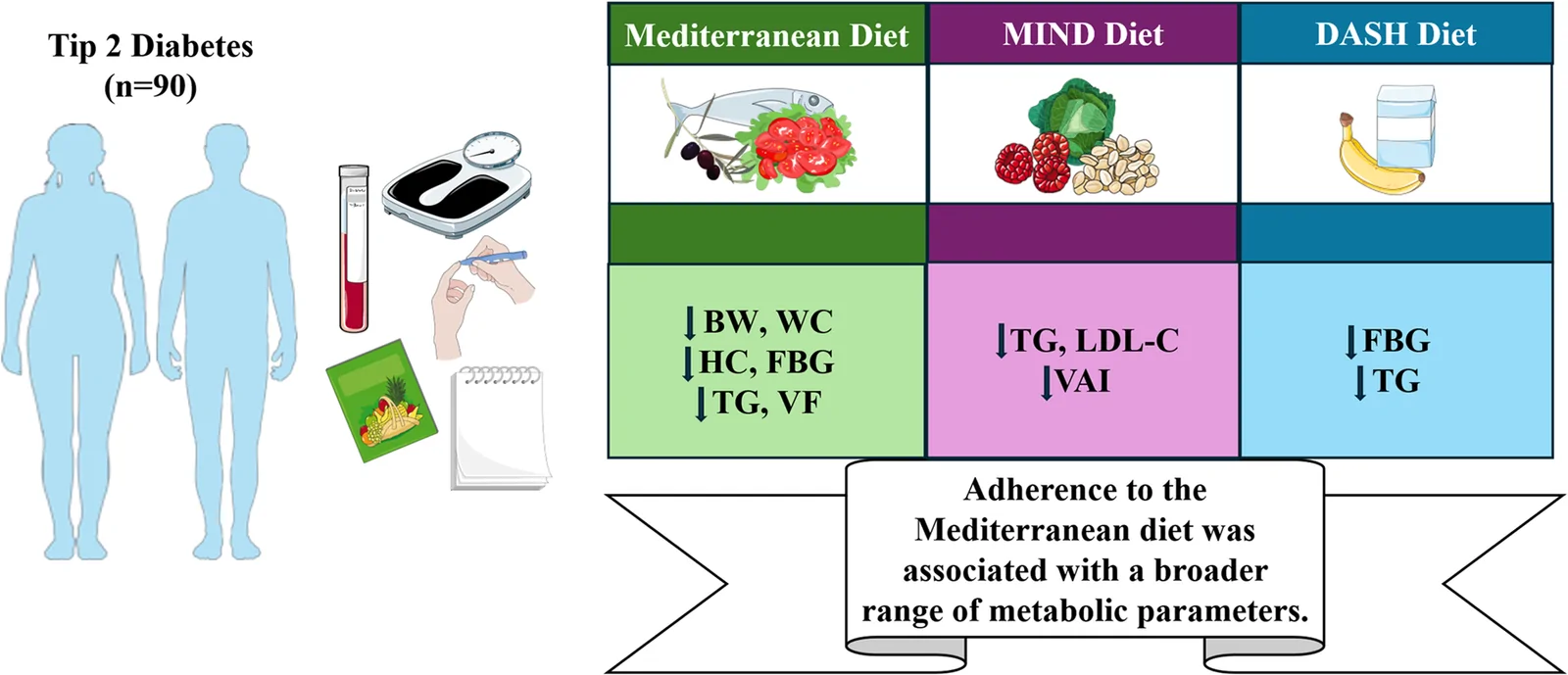
This figure was created using images from Servier Medical Art (https://smart.servier.com), which are licensed under a Creative Commons Attribution 4.0 Unported License. Abbreviations: MIND (Mediterranean–DASH intervention for neurodegenerative delay), DASH (dietary approaches to stop hypertension), BW (body weight), WC (waist circumference), HC (hip circumference), FBG (fasting blood glucose), TG (triglycerides), VF (visceral fat), VAI (visceral adiposity index), and LDL-C (low-density lipoprotein cholesterol)

## Introduction

Type 2 Diabetes Mellitus (T2DM) is a condition characterized by long-term high blood sugar (hyperglycemia) and high insulin levels (hyperinsulinemia) [1]. DM, particularly T2DM, is placing an increasing burden on healthcare systems worldwide [2]. According to data from the Diabetes Atlas 2025 published by the International Diabetes Federation (IDF), approximately 589 million adults worldwide are living with diabetes, and this number is expected to reach 853 million by 2050 [3]. The development of T2DM is closely linked to various metabolic health indicators. Therefore, diabetes-related biomarkers are clinically important for early risk prediction and improving disease progression. Fasting blood glucose (FBG), body mass index (BMI), high-density lipoprotein cholesterol (HDL-C), hemoglobin A1c (HbA1c), triglycerides (TG), Visceral Adiposity Index (VAI), total cholesterol (TC), and waist circumference (WC) are frequently used in the assessment of metabolic status [4]. Monitoring these parameters is crucial due to the numerous metabolic and homeostatic disorders that arise as the disease progresses. Impairments in glucose and lipid metabolism weaken vascular integrity, negatively affecting blood flow and consequently leading to organ dysfunction and an increased risk of early mortality [5]. Rapid urbanization and modernization have led to permanent changes in lifestyle components such as unhealthy eating habits, insufficient physical activity, increased stress, and environmental factors. These changes facilitate the emergence of metabolic disorders, leading to the increasing prevalence of T2DM worldwide [6].

Lifestyle factors such as diet play a fundamental role in T2DM management. In recent years, the effects of different dietary patterns on T2DM management have been investigated [7]. One study found that a diet low in animal products and high in plant-based foods was associated with reduced insulin resistance and lower risks of prediabetes and T2DM. This association remained significant even after adjusting for body weight (BW) [8]. The literature highlights the importance of recommending plant-based diets in nutrition guidelines to reduce the burden of T2DM [9]. Dietary patterns that place a strong emphasis on plant-derived foods include the Dietary Approaches to Stop Hypertension (DASH) diet, the Mediterranean diet, and the Mediterranean–DASH Intervention for Neurodegenerative Delay (MIND) diet [10]. The Mediterranean Diet is a plant-based eating pattern rich in plant foods, using olive oil as the primary fat source, featuring low red meat, moderate fish and dairy products, and moderate wine consumption with meals [11]. Adherence to the Mediterranean diet has been associated with a reduced incidence of T2DM and improved glycemic control in individuals with the disease [12, 13]. The DASH diet is widely recommended for blood pressure control and has been associated with reductions in blood pressure (BP). The diet includes plenty of vegetables, fruits, and low-fat dairy products, while also incorporating fish, nuts, and legumes with moderate sodium restriction [14]. Higher levels of adherence to the DASH diet are associated with significant improvements in lipid profiles, particularly through reductions in TC and LDL-C levels [15, 16]. The MIND diet is a dietary model developed by adapting the key features of the Mediterranean and DASH diets to focus on cognitive health. The diet emphasizes increasing the consumption of plant-based foods, such as green leafy vegetables and fruits, while limiting animal-based and saturated fat-rich foods [17]. In a study conducted in obese individuals, the relationship between adherence to the MIND diet and cardiometabolic markers was examined; a significant relationship was only found in terms of insulin sensitivity [18]. A significant limitation in this field is the scarcity of studies that simultaneously examine the relationships between adherence to different healthy dietary patterns and metabolic parameters [12, 13, 15, 16, 18]. Given this gap in the literature, this study was designed to examine the relationships between adherence to the DASH, Mediterranean, and MIND diets and various metabolic parameters in individuals with type 2 diabetes. In this context, it was hypothesized that adherence to these dietary patterns may be associated with metabolic parameters and that these relationships may vary depending on the specific dietary pattern.

## Materials and methods

### Participants and study design

The study population consisted of individuals diagnosed with T2DM in accordance with the diagnostic criteria of the American Diabetes Association [19], who applied to the Internal Medicine Outpatient Clinic of Bolu Abant Izzet Baysal University (BAIBU) Training and Research Hospital. For this cross-sectional study, the required sample size was determined using the G*Power 3.1 statistical software. In the sample calculation, a 5% significance level (α = 0.05), 80% statistical power (1 − β = 0.80), and a medium effect size (f² = 0.15) capable of revealing the relationship between metabolic parameters and diet scores were assumed. Based on the calculation performed using multivariate linear regression analysis, the minimum sample size required for the study was determined to be 85 individuals, and the study was completed with 90 individuals with T2DM. Individuals with severe physical or psychiatric conditions, including bipolar disorder, schizophrenia, kidney disease or end-stage liver, chronic inflammatory disorders, amputations, and neoplastic diseases, as well as those who declined to participate, were excluded from the study.

Before enrollment, written informed consent was obtained from all participants. The study protocol was approved by the Clinical Research Ethics Committee of BAIBU (Protocol No. 2023/451) and registered in the ClinicalTrials.gov database (NCT07310225).

### Data collection

#### Questionnaire

Participants’ sociodemographic characteristics (gender, age, alcohol and smoking use, presence of disease) were inquired.

#### Anthropometric measurements

Within the scope of anthropometric measurements of individuals, body weight (BW), WC, height (Ht), and hip circumference (HC), were measured; accordingly, BMI was calculated. BW was measured using a calibrated digital scale with 100-g precision, placed on a level, non-slippery tiled floor. Ht was assessed with a Dikomsan stadiometer, with participants standing upright, feet together, with the head aligned in the Frankfort horizontal position. WC was assessed at the midpoint between the inferior margin of the last rib and the iliac crest, whereas HC was measured at the widest part of the hips. BMI was calculated as BW divided by height squared (kg/m²).

#### Body composition

Body composition measurements (fat mass (FM) (%), visceral fat (VF) (%), basal metabolic rate (BMR) (kcal), fat-free mass (FFM) (kg), total body water (TBW) (%)) were obtained using a bioelectrical impedance analysis (BIA) device (Tanita Innerscan BC 401, Japan). BIA measurements were performed under standard conditions to increase the reliability of the results. Measurements were preferably taken in the morning, with individuals having fasted for at least 8–12 h. During the measurement, participants were evaluated wearing light clothing and barefoot, with metal accessories removed. For standing BIA measurements, participants were instructed to stand upright without locking their knees and without their arms touching their body. Measurements were performed using the same device and protocol. For female participants, the possibility that the menstrual cycle could affect fluid distribution was taken into account. Participants were recruited during routine clinical visits, and no specific instructions were provided regarding avoidance of vigorous physical activity, alcohol, or caffeine intake prior to BIA measurements. In addition, bladder emptying was not standardized before the assessment.

#### Biochemical parameters

The blood parameters requested for routine outpatient follow-up of diabetic individuals (FBG, HbA1c, TG, LDL-C, HDL-C) were retrieved from the hospital’s electronic data management system.

#### Visceral adiposity index (VAI)

VAI has been used in the assessment of abdominal obesity. In the VAI assessment, individuals’ WC, TG, BMI, and HDL-C values were used. The calculation was performed according to gender as follows [20]:

$$\begin{array}{c}\mathrm{VAI}\;(\mathrm{Female})\;=\;\lbrack\mathrm{WC}\;(\mathrm{cm})/\;(36.58+(1.88\times\mathrm{BMI})\rbrack\\\times(\mathrm{TG}/0.81)\times(1.52/\mathrm{HDL}-\mathrm C),\\\mathrm{VAI}\;(\mathrm{Male})\;=\;\lbrack\mathrm{WC}\;(\mathrm{cm})/\;(36.58+(1.88\times\mathrm{BMI})\rbrack\\\times(\mathrm{TG}/1.03)\times(1.31/\mathrm{HDL}-\mathrm C).\end{array}$$

#### Nutritional status

A food frequency questionnaire and food intake record were used to assess individuals’ nutritional status. The Mediterranean Diet Scale, DASH diet adherence score, and MIND diet adherence scale were used to calculate the nutritional pattern scores.

#### Food frequency questionnaire

Dietary intake was assessed using a Food Frequency Questionnaire that evaluated foods consumed over the previous month. The questionnaire included seven food groups: five basic food groups (vegetables-fruits, bread-grains, milk-dairy products, sugars-fats, meat-meat products), as well as beverages and fast food. Participants reported both the frequency and portion sizes of consumed foods using eight predefined response options: “every day,” “at each meal,” “three to four times weekly,” “five to six times weekly,” “one to two times weekly,” “once a month,” “once every two weeks,” and “not consumed.” To estimate average daily intake, reported consumption frequencies were converted into daily equivalents using standardized coefficients. Portion sizes were multiplied by these coefficients to calculate mean daily food and beverage intakes. Daily energy, macronutrient, and micronutrient intakes were subsequently calculated based on these values.

#### Food consumption record

A single-day food consumption record was obtained using the 24-hour recall method to determine participants’ food consumption patterns. To ensure accurate and reliable assessment of food quantities consumed, portion estimates were made using the Food and Nutrition Photo Catalog [21]. The assessment of daily energy and nutrient intake was performed through the Nutrition Information System (BEBİS), using data derived from individuals’ reported food intake over the preceding 24 h. The combined assessment of food consumption frequency and the 24-hour dietary record increased the validity of the data obtained by supporting short-term intake with long-term dietary patterns.

#### DASH diet compliance score

The diet score is based on a total of 8 food groups: vegetables, fruits, grains, nuts, low-fat dairy products, seeds and legumes, lean meats, oils and fatty seeds, and sweets and added sugars. For each food group, the recommended portion sizes determined according to individuals’ daily energy intake levels (1600, 2000, 2600, and 3100 kcal/day) were used as a basis. Individuals who fully complied with the recommended portions received 1 point, those who partially complied received 0.5 points, and those who fell below or above the recommendations received 0 points. Individuals’ DASH diet compliance scores were calculated based on their food consumption frequencies after their energy requirements were calculated. The maximum score that could be obtained on the DASH diet compliance scale was 8.0, with scores of 4.5 and above being considered ‘high compliance’ and scores below 4.5 being considered ‘low compliance’ [22].

#### Mediterranean diet adherence scale

The Mediterranean Diet Scale was used to determine individuals’ adherence to the Mediterranean diet. This scale consists of a total of 14 questions, 2 of which relate to food consumption habits and 12 of which relate to food consumption frequency. Each question is scored as 0 or 1. The scale’s scoring range is set at 0–14 points. The validity and reliability study of the Turkish version of the scale, originally developed by Martínez-Gonzalez et al. [23], was conducted by Pehlivanoğlu et al. [24]. A score of 0–5 indicates the lowest compliance, 6–9 indicates moderate compliance, and ≥ 10 indicates high compliance with the Mediterranean diet.

#### MIND diet adherence scale

This scale consists of 15 food groups, including ten groups considered protective for brain health and five groups regarded as detrimental. Dietary intake was evaluated using food consumption records and Food Frequency Questionnaire data, and the amounts of foods consumed from these 15 groups were converted into portion sizes and scored according to the predefined scoring criteria as originally described by Morris et al. [25]. The total score ranges from 0 to 15, with no specific cutoff value for defining adherence; higher scores indicate greater adherence to the MIND diet. The validity and reliability of the Turkish version of the scale have been established by Atabilen et al. [26].

Statistical analyses were performed using IBM SPSS Statistics software (version 25.0; IBM Corp., Armonk, NY, USA), and a two-sided significance level of *p* < 0.05 was applied. Measures of central tendency and variability (mean, standard deviation, and median) were used to describe continuous data, whereas categorical variables were summarized as counts and proportions. Data distribution was assessed using Kolmogorov–Smirnov and Shapiro–Wilk tests. Depending on distribution characteristics, between-group differences were analyzed using either the independent samples t-test or the Mann–Whitney U test. Associations between dietary pattern scores and metabolic parameters were examined through correlation analyses performed in RStudio, and the resulting correlation coefficients were graphically represented using heatmap visualization. Radar charts were created using the absolute values of standardized beta (β) coefficients obtained from multiple regression analyses to illustrate the associations of MIND, Mediterranean, and DASH diet adherence scores with metabolic parameters.

## Results

The general characteristics of female and male participants are presented in Table 1. The mean age of female participants in the study was 57.97 ± 7.88 years, while the mean age of male participants was 56.39 ± 11.94 years (*p* > 0.05). It was observed that most female and male participants had diseases other than diabetes. It was found that the vast majority of female and male participants did not use tobacco or alcohol. HbA1c (%), TG, and LDL-C levels did not show significant differences according to gender. Female’s HDL-C levels were 58.05 ± 12.10, while male’s were 38.28 ± 1.89 (*p* < 0.001). Female’s FM levels were significantly higher than male’s, while their FFM levels were lower (*p* < 0.05). No significant difference was observed between female and male in terms of BW, WC, HC, BMI, BMR, and VAI levels. Male’s protein intake was significantly higher than female’s (*p* < 0.05). No statistically significant differences were found between male and female participants with respect to MIND, Mediterranean, and DASH diet scores, as well as total energy, carbohydrate, and fat intakes.

**Table 1:** General characteristics of female and male with type 2 diabetes

Variables	Female Mean ± SD / (*n*, %)	Male Mean ± SD / (*n*, %)	*p*
Age	57.97 ± 7.88	56.39 ± 11.94	0.534
Presence of illness			
Yes	27 (79.4%)	19 (73.1%)	0.565
No	7 (26.4%)	7 (28.9%)	
Smoking status			
Smoker	9 (20.5%)	2 (6.3%)	0.082
Non-smoker	35 (79.5%)	30 (93.8%)	
Alcohol consumption			
Current drinker	0 (0%)	2 (6.3%)	0.340
Non-drinker	44 (100%)	30 (93.8%)	
FBG (mg/dL)	131.96 ± 59.31	115.15 ± 70.08	**0.032**
HbA1c (%)	8.26 ± 2.04	7.21 ± 2.44	0.061
TG (mg/dL)	122.26 ± 52.74	122.14 ± 54.81	0.993
LDL-C (mg/dL)	133.02 ± 19.07	117.07 ± 46.64	0.094
HDL-C (mg/dL)	58.05 ± 12.10	38.28 ± 1.89	**< 0.001**
BW (kg)	82.61 ± 14.37	77.91 ± 15.19	0.189
WC (cm)	105.36 ± 11.72	103.51 ± 7.01	0.431
HC (cm)	109.24 ± 12.18	106.08 ± 7.10	0.203
BMI (kg/m²)	30.78 ± 6.08	28.50 ± 4.20	0.060
FM (%)	42.67 ± 7.00	35.43 ± 7.65	**< 0.001**
FFM (kg)	43.61 ± 5.79	47.12 ± 6.00	**0.018**
BMR (kkal)	1447.92 ± 159.21	1497.11 ± 149.79	0.194
VAI	5.±59 ± 1.35	5.32 ± 1.58	0.557
Diet score			
MIND	6.67 ± 1.24	6.43 ± 1.21	0.410
Mediterranean	4.66 ± 1.67	4.00 ± 1.38	0.064
DASH	1.34 ± 0.74	1.33 ± 0.54	0.963
Energy intake (kcal/day)	980.51 ± 288.35	1126.86 ± 512.34	0.163
Carbohydrate intake (%/day)	61.06 ± 10.48	58.90 ± 14.71	0.835
Protein intake (%/day)	11.56 ± 3.30	14.06 ± 4.16	**0.005**
Fat intake (%/day)	27.27 ± 8.98	26.70 ± 14.52	0.461

*FBG* fasting blood glucose, *HbA1c* hemoglobin A1c, *TG* triglyceride, *LDL-C* low-density lipoprotein cholesterol, *HDL-C* high-density lipoprotein cholesterol, *BW* body weight, *WC* waist circumference, *HC* hip circumference, *BMI* body mass index, *FM* fat mass, *FFM* fat-free mass, *BMR* basal metabolic rate, *VAI* visceral adiposity index, *MIND* Mediterranean–DASH diet intervention for neurodegenerative delay, and *DASH* dietary approaches to stop hypertension

The independent samples t-test was applied to continuous data, and categorical variables were evaluated using the chi-square test

Statistically significant results are highlighted in bold (*p* < 0.05)

Anthropometric and biochemical parameters according to participants’ adherence to the Mediterranean, DASH, and MIND diets are presented in Table 2. It was observed that those with low adherence to the Mediterranean diet had significantly higher WC and FM levels (*p* < 0.05). No significant difference was observed in terms of BW and VF between those with low and moderate adherence to the Mediterranean diet. Those with low adherence to the Mediterranean diet were found to have significantly higher FBG and TG levels (*p* < 0.05). No significant difference was observed between the groups in terms of LDL-C and VAI levels. No significant difference was observed in terms of BW, WC, FM, and VAI levels between those with low and high adherence to the DASH diet. It was found that those with low adherence to the DASH diet had significantly higher FBG and TG levels compared to those with high adherence (*p* < 0.05). No significant differences were observed in BW, WC, FM, VF, FBG, and LDL-C levels between those with low and high adherence to the MIND diet. Participants with low adherence to the MIND diet were found to have significantly higher TG and VAI levels compared to those with high adherence (*p* < 0.05).

**Table 2:** Anthropometric and biochemical parameters according to adherence to the Mediterranean, DASH, and MIND diets in individuals with type 2 diabetes

Variables	Mediterranean diet		p1	DASH diet		p2	MIND diet		p3
	Low adherence	Moderate adherence		Low adherence	High adherence		Low adherence	High adherence	
BW (kg)	81.14 ± 16.02	75.50 ± 11.49	0.222	82.50 ± 15.25	74.75 ± 13.36	0.060	80.26 ± 16.62	79.00 ± 13.21	0.717
WC (cm)	105.85 ± 10.29	100.94 ± 8.77	**0.045**	105.87 ± 9.52	101.27 ± 10.77	0.098	104.77 ± 10.40	104.37 ± 9.69	0.875
FM (%)	39.21 ± 7.79	35.19 ± 8.82	**0.032**	39.28 ± 7.89	36.29 ± 8.44	0.139	38.26 ± 8.28	38.15 ± 8.17	0.080
VF (%)	6.95 ± 2.73	5.75 ± 2.98	0.109	7.00 ± 2.83	5.94 ± 2.66	0.112	6.56 ± 2.90	6.72 ± 2.74	0.821
FBG (mg/dL)	129.51 ± 58.74	100.66 ± 54.09	**<0.001**	125.95 ± 54.87	98.55 ± 52.91	**0.020**	122.41 ± 65.43	101.33 ± 63.53	0.225
LDL-C (mg/dL)	127.05 ± 34.54	129.84 ± 29.50	0.755	128.50 ± 33.43	124.19 ± 32.67	0.595	131.90 ± 34.87	121.35 ± 30.15	0.166
TG (mg/dL)	133.45 ± 44.93	102.21 ± 35.07	**0.008**	133.87 ± 41.68	100.69 ± 39.15	**0.024**	137.35 ± 40.21	104.41 ± 51.13	**0.010**
VAI	5.63 ± 1.53	4.93 ± 1.38	0.139	5.57 ± 1.54	4.87 ± 1.31	0.163	5.94 ± 1.55	4.65 ± 1.08	**0.001**

*BW* body weight, *WC* waist circumference, *FM* fat mass, *VF* visceral fat, *FBG* fasting blood glucose, *TG* triglyceride, *LDL-C* low-density lipoprotein cholesterol, and *VAI* visceral adiposity index

Group comparisons were performed using the independent samples t-test for normally distributed variables, while the Mann–Whitney U test was employed for variables showing non-normal distributions

Statistically significant results are highlighted in bold (p < 0.05). p1 represents the comparison according to the Mediterranean diet, p2 according to the DASH diet, and p3 according to the MIND diet

**Fig. 1:**
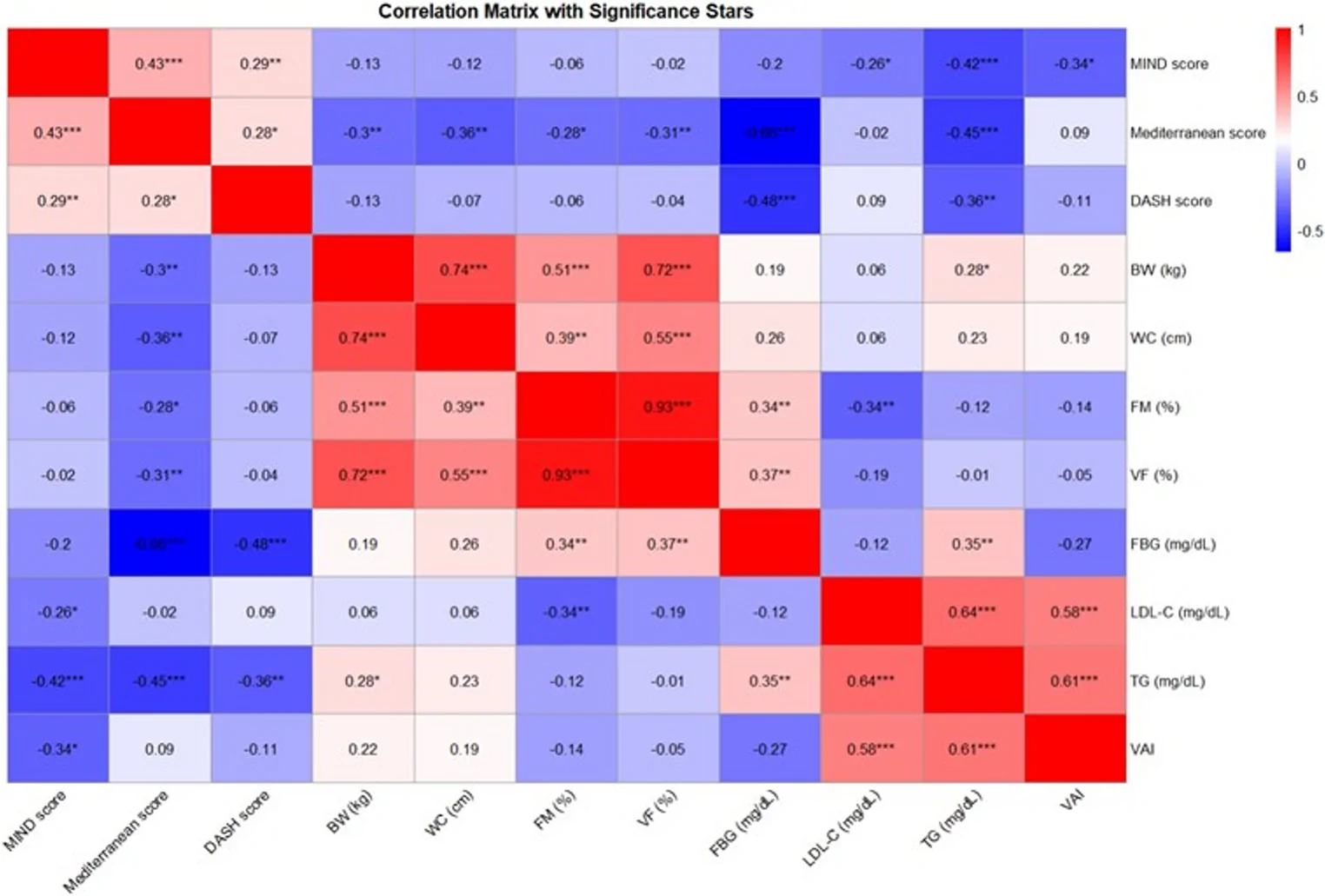
Heatmap depicting correlation coefficients between adherence scores for the MIND, Mediterranean, and DASH dietary patterns and selected anthropometric and biochemical parameters in individuals with type 2 diabetes. The matrix summarizes the relationships between diet scores and clinical indicators, including BW, WC, FM, VF, FBG, LDL-C, TG, and VAI. The magnitude and direction of the correlations are represented by color gradients, with blue denoting inverse associations and red indicating positive associations. Statistical significance was defined using the following thresholds: *p < 0.05, **p < 0.01, and ***p < 0.001

The correlation relationships between MIND, Mediterranean, and DASH diet scores and anthropometric and biochemical variables in individuals with type 2 diabetes are presented in Fig. 1. A negative correlation was observed between participants’ MIND diet scores and LDL-C, TG, and VAI levels (*r*=-0.26, *r*=-0.42, *r*=-0.34). A negative correlation was found between the Mediterranean diet score and BW, WC, FM, VF, FBG, and TG levels (*r*=-0.30, *r*=-0.36, *r*=-0.28, *r*=-0.31, *r*=-0.66, *r*=-0.45). A negative correlation was found between participants’ DASH diet scores and FBG and TG levels (*r*=-0.48, *r*=-0.36). As individuals’ Mediterranean diet scores increased, their MIND and DASH diet scores also increased.

The results of regression analyses on metabolic parameters according to adherence to three different dietary models in individuals with type 2 diabetes are presented in Table 3. In Model 1, each 1-point increase in the MIND score was associated with a decrease of 0.011 mg/dL, 0.010 mg/dL, and 0.387 units in LDL-C, TG, and VAI values, respectively. In Model 2, each 1-point increase in the Mediterranean diet score was associated with a decrease of 0.028, 0.058, 0.043, and 0.139 units in BW, WC, HC, and VF, respectively. Additionally, decreases of 0.016 and 0.014 units in FBG and TG levels, respectively, were observed. The Mediterranean diet score explained 11.1%, 14.9%, 6.0%, 7.5%, 49.4%, and 30.1% of the variance in BW, WC, HC, VF, FBG, and TG variables, respectively. In Model 3, each 1-point increase in the DASH diet score was associated with a decrease of 0.007 and 0.005 units in FBG and TG levels, respectively. The DASH diet score explained 25.2% and 11.9% of the variance in FBG and TG, respectively.

**Table 3:** Regression analysis results of metabolic parameters according to adherence to three different dietary patterns in individuals with type 2 diabetes

Model 1	BW (kg)		WC (cm)		HC (cm)		VF (%)		FBG (mg/dL)		LDL-C (mg/dL)		TG (mg/dL)		VAI	
	β	ΔR^2^	β	ΔR^2^	β	ΔR^2^	β	ΔR^2^	β	ΔR^2^	β	ΔR^2^	β	ΔR^2^	β	ΔR^2^
MIND score	-0.015	0.041	-0.020	0.028	-0.015	0.014	0.002	0.004	-0.004	0.031	-0.011*	0.079*	-0.010***	0.170***	-0.387**	0.167**
Model 2																
Mediterranean score	-0.028*	0.111*	-0.058**	0.149**	-0.043*	0.060*	-0.139*	0.075*	-0.016***	0.494***	-0.005	0.054	-0.014***	0.301***	0.078	0.046
Model 3																
DASH score	-0.005	0.015	-0.001	0.035	-0.007	0.057	-0.005	0.020	-0.007***	0.252***	0.002	0.027	-0.005**	0.119**	-0.005	0.080

DASH (dietary approaches to stop hypertension), MIND (Mediterranean–DASH intervention for neurodegenerative delay), VAI (visceral adiposity index), BW (body weight), WC (waist circumference), HC (hip circumference), VF (visceral fat), FBG (fasting blood glucose), LDL-C (low-density lipoprotein cholesterol), and TG (triglycerides). Linear regression analyses were conducted to assess statistical significance, with all models adjusted for age and sex. Separate regression models were generated for each metabolic outcome as follows: Model 1 incorporated the MIND diet score, Model 2 included the Mediterranean diet score, and Model 3 was based on the DASH diet score. ***p < 0.05, **p < 0.01, and *p < 0.001 indicate two-tailed levels of statistical significance

**Fig. 2:**
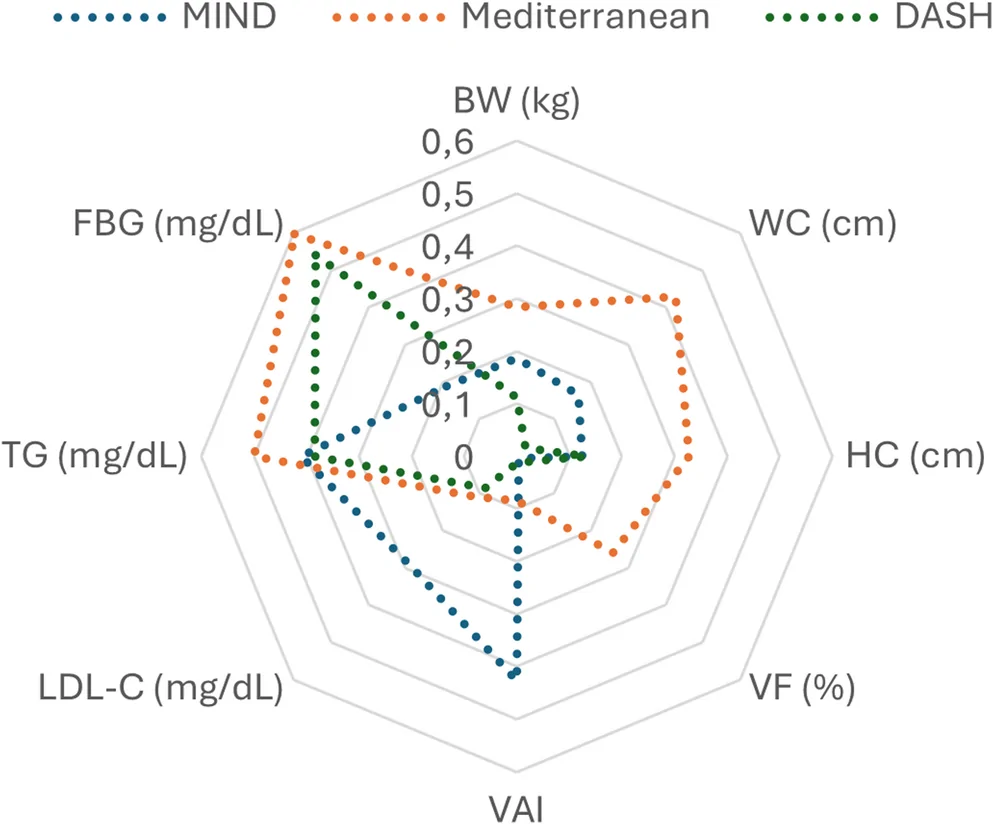
Radar plot showing the absolute values of standardized beta (β) coefficients for the relationships between metabolic parameters and MIND, Mediterranean, and DASH diet scores in individuals with type 2 diabetes. Higher absolute β coefficients reflect stronger associations with the relevant metabolic parameters. Adherence to the MIND diet was found to be associated with TG and VAI, while adherence to the Mediterranean diet was associated with FBG, LDL-C, and VF. Adherence to the DASH diet is also associated with various metabolic parameters; however, this figure does not show a direct comparison or superiority among these dietary patterns

Figure 2 shows the absolute values of the standardized beta (β) coefficients of the Mediterranean, DASH, and MIND diet scores on metabolic parameters. For the MIND diet, higher standardized β coefficients were observed for TG and VAI. In the Mediterranean diet, relatively higher β coefficients were noted for FBG, LDL-C, and VF. The DASH diet showed generally lower β coefficients across the examined parameters.

## Discussion

In this study, it was hypothesized that adherence to the Mediterranean, DASH, and MIND diets in individuals with type 2 diabetes might be associated with various metabolic parameters, and that these associations might differ depending on the dietary pattern. The findings indicate that adherence to these dietary patterns is associated with markers of metabolic health. In particular, it was observed that higher adherence to the Mediterranean diet was associated with a greater number of metabolic parameters. Analyses revealed that, after adjusting for confounding variables, adherence to the MIND diet was inversely associated with LDL-C, TG, and VAI; adherence to the Mediterranean diet was inversely associated with BW, WC, HC, FBG, LDL-C, and TG; and adherence to the DASH diet was inversely associated with FBG and TG. Overall, adherence to the Mediterranean diet appears to be associated with a greater number of metabolic parameters.

Evidence from systematic reviews suggests that high adherence to the Mediterranean diet is associated with beneficial outcomes in relation to metabolic and cardiovascular complications [27]. In individuals with T2DM, moderate carbohydrate restriction (< 45% of total energy) has been reported to reduce TG concentrations by − 0.13 mmol/L [28], whereas Mediterranean diet implementation reduces TG by − 0.41 mmol/L [29], based on moderate-quality evidence. In the ATTICA cohort study, individuals with low adherence to the Mediterranean diet showed certain changes in lipid profiles, including elevated TG levels, which were associated with increased cardiovascular disease risk [30]. In a non-randomized study, a 12-week Mediterranean diet intervention was associated with reductions in FBG, TG, and BP, and an increase in HDL-C, compared with the control group [31]. Evidence from previous studies [28–31] suggests an inverse association between adherence to the Mediterranean diet and FBG and TG levels, which is broadly consistent with the associations observed in the present cross-sectional study. Furthermore, a higher level of adherence to the Mediterranean diet was found to be significantly and inversely associated with levels of BW, FM, and VF, suggesting that it may be associated with improved glycemic control and reduced adiposity in individuals with type 2 diabetes.

Following DASH diet interventions in overweight and obese individuals, no significant changes were observed in biochemical parameters; however, reductions in BW, WC, FM, and VF were reported [32]. One study showed that adherence to the DASH diet was associated with lower values for BW, diastolic and systolic blood pressure (DBP and SBP), TC, and LDL-C levels compared to the control group, while no significant changes were observed in FBG, HDL-C, TG, HOMA-IR, and C-reactive protein levels. Taken together, these findings suggest that adherence to the DASH diet may have potential beneficial effects on major cardiovascular disease (CVD) risk factors; however, its ultimate impact on CVD incidence and clinical outcomes remains unclear [33]. A meta-analysis including randomized controlled trials showed that adherence to the DASH dietary pattern was linked to reductions in SBP and DBP, LDL-C, and TC levels in individuals with normal BP or stage 1 hypertension compared to the control group [34]. In this study, after adjusting for significant covariates, adherence to the DASH diet was not associated with anthropometric indicators such as BW and WC, but was inversely associated with FBG and TG levels (Figs. 1 and 2; Table 3). These findings suggest that the associations between the DASH diet and metabolic parameters may vary across different populations and may be more evident for lipid and glycemic parameters, particularly in individuals with T2DM. On the other hand, a study reported no significant association between the DASH diet score and VAI [35]. Similar findings were obtained in this study as well, and no significant association was observed between adherence to the DASH diet and visceral fat accumulation.

A study found that the lowest CV mortality risk was observed in individuals without diabetes who had a high MIND score. In contrast, it was reported that CV mortality risk gradually increased in individuals with low MIND scores, particularly in the presence of diabetes [36]. Furthermore, while no significant relationship was found between the MIND diet score and FBG, HbA1c, and LDL-C levels in the aforementioned study [36], our study found that adherence to the MIND diet was inversely associated with LDL-C and TG levels. These differences may stem from the characteristics of the study populations, sample sizes, statistical models used, and the varying approaches to lipid-lowering drug use in the studies. Consistent with our findings, higher MIND diet scores have been reported to be associated with lipid parameters indicative of a healthier profile in individuals with steatotic liver disease [37]. A study conducted on 203 overweight/obese adolescents reported an inverse association between MIND diet scores and the likelihood of hyperglycemia and insulin resistance [38]. High adherence to the MIND diet was associated with a lower likelihood of having the metabolically unhealthy overweight/obese (MUO) phenotype [38]. In contrast, in our sample of 90 adults with type 2 diabetes, no significant association was observed between adherence to the MIND diet and fasting blood glucose, body weight, or waist circumference; however, an inverse association was found with VAI (Fig. 1). The dominant effect of medication on fasting blood glucose levels, which influences glycemic control in adults with type 2 diabetes, may have masked potential associations between diet and glycemic parameters.

There are few studies in the literature that examine the Mediterranean, DASH, and MIND dietary patterns together. In the systematic review, the DASH diet was examined alongside standard and various experimental dietary approaches; however, the Mediterranean and MIND diets were not included among the analyzed studies. The review reported positive associations between the DASH diet and metabolic syndrome components such as SBP, DBP, HDL-C, and LDL-C [39]. In a cross-sectional study examining the associations between plant-based diets and cardiometabolic risk, adherence to the Mediterranean diet was found to be associated with more favorable outcomes regarding glucose metabolism and blood pressure, while the DASH diet was found to be associated with a particularly favorable lipid profile. The MIND diet stands out in terms of vascular function and long-term CV risk due to its positive relationship with flow-mediated dilation and its inverse correlation with the 10-year atherosclerotic CVD risk score [40]. The DASH, Mediterranean, and MIND diets have been shown to be associated with cardiometabolic health in various ways. In a study examining these three dietary patterns simultaneously in adults, it was reported that high adherence to the Mediterranean diet was inversely associated with FBG, high adherence to the DASH diet was inversely associated with WC, and high adherence to the MIND diet was inversely associated with SBP [41]. The relationships between adherence to four dietary indices (MIND, HEI-2015, DASH, and Mediterranean) and markers of metabolic health and obesity in adults were examined. After adjusting for covariates, the MIND diet emerged as one of the dietary indices showing significant associations with components of metabolic syndrome and visceral obesity. These findings suggest that the relationships between the MIND diet and other healthy dietary patterns may differ in terms of metabolic health markers and that these relationships may be linked to mechanisms that protect cognitive function [42]. In our study, higher standardized beta coefficients were observed for the Mediterranean diet in relation to certain metabolic parameters, while the DASH and MIND diets showed relatively lower coefficients (Fig. 2; Table 3); this suggests that the relationships with FBG, LDL-C, and VF may vary to different degrees. This difference may stem from individuals with T2DM showing higher adherence to the Mediterranean diet and the diet being a flexible model that is compatible with cultural eating habits and widely recommended in diabetes nutrition education. In contrast, the MIND diet is a more targeted and limited dietary model that emphasizes the regular consumption of green leafy vegetables and specific foods such as strawberries and blueberries. This may have made adherence to the MIND diet more difficult in daily practice and led to more limited relationships with metabolic parameters. In a study conducted on adults with high normal BP or stage 1 hypertension, the simultaneous application of the DASH diet and salt restriction reduced HbA1c and BP compared to salt restriction alone. The Mediterranean diet, in addition to these, also led to reductions in FBG, TC, and LDL-C levels [43]. In our study, adherence to the Mediterranean diet was found to be associated with certain metabolic parameters in ways that differ from the associations observed with the DASH and MIND diets (Figs. 1 and 2; Table 3). While this finding appears consistent with the results of the intervention study reported in the literature [43], causal inferences cannot be drawn due to the observational nature of our study.

Among the strengths of this study are the observational examination of the relationships between metabolic parameters and dietary habits in patients with T2DM, the comprehensive assessment of nutritional status using validated methods, and the inclusion of multiple dietary indices (the Mediterranean diet, the DASH diet, and the MIND diet) within the same analytical framework. Additionally, the combined evaluation of anthropometric measurements, biochemical parameters, and body composition indicators has enabled the adoption of a multidimensional approach to metabolic health. The use of multivariate and hierarchical linear regression analyses, which include statistical adjustments for potential confounding variables, is another key element that enhances the study’s methodological strength.

However, the study has some limitations. The cross-sectional and descriptive design does not allow the findings to be interpreted as causal relationships. Since the sample consists of a limited number of T2DM patients from a single center, the generalizability of the results to broader populations may be limited. Furthermore, while the sample size is sufficient for the current analyses, it may create limitations in terms of external validity when compared to studies conducted with larger and more heterogeneous samples. Another significant limitation is that dietary data were self-reported, and diet adherence scores were calculated post-hoc without intervention; this may affect the interpretation of relationships between diet and metabolic parameters. Therefore, prospective studies with larger samples are necessary to validate the findings and enhance generalizability. In addition, the lack of standardization of pre-measurement conditions for BIA (e.g., physical activity, alcohol and caffeine intake, and bladder status) may have influenced body composition measurements.

In conclusion, adherence to the Mediterranean, DASH, and MIND diets was found to be associated with metabolic parameters in adults with T2DM. Adherence to the Mediterranean diet was associated with different levels of association with certain parameters compared to the other dietary models. These findings highlight the potential role of adherence to healthy dietary patterns in metabolic health and may be considered in the development of diabetes management and dietary recommendations from a public health perspective. Prospective studies are needed to generalize these findings to broader populations and to clarify causal relationships more clearly.
